# SARS-CoV-2 reinfection with Omicron variant in Shaanxi Province, China: December 2022 to February 2023

**DOI:** 10.1186/s12889-024-17902-6

**Published:** 2024-02-16

**Authors:** Mengyan Zhang, Lei Cao, Luqian Zhang, Xinxin Li, Sa Chen, Yi Zhang

**Affiliations:** Shaanxi Provincial Centre for Disease Prevention and Control, Xi’an, People’s Republic of China

**Keywords:** SARS-CoV-2, COVID-19, Reinfection

## Abstract

**Background:**

Prior to December 2022, there were no reports of reinfection with the severe acute respiratory syndrome coronavirus 2 (SARS-CoV-2) in Shaanxi province, China. Since then, China has refined its strategy in response to coronaviruses. The purpose of this study was to determine the incidence of SARS-CoV-2 reinfections and its contributing factors, as well as to compare clinical characteristics between first and second episodes of infection in Shaanxi Province, China between December 2022 and February 2023.

**Methods:**

We conducted a cross-sectional study using an epidemiological survey system and electronic questionnaires to investigate the incidence of SARS-CoV-2 reinfection among previously infected individuals during the epidemic wave owing to the Omicron variant that began in December 2022. A logistic regression model was used to determine those factors influencing SARS-CoV-2 reinfections.

**Results:**

According to the virus variant that caused the first infection, the rate of reinfection for the Omicron variants was 1.28%, 1.96%, and 5.92% at 2–3 months, 4–5 months, and 7–9 months after the primary infection, respectively. The rate of reinfection for the Delta variants was 25.10% 11–12 months after the primary infection. Females, adults between 18 and 38 years and being a medical worker were associated with an increased risk of reinfection. Fever, cough, sore throat and fatigue were the four most common clinical symptoms during both first and second COVID-19 infections.

**Conclusions:**

In our study, the rate of SARS-CoV-2 reinfection increased over time during epidemic waves predominantly involving the Omicron variant in Shaanxi province, China. Large-scale infections are less likely in subsequent Omicron epidemic waves. Nevertheless, it is essential to continuously monitor cases of infection as well as continue surveillance for emerging SARS-CoV-2 variants.

## Background

The first confirmed case of severe acute respiratory syndrome coronavirus 2 (SARS-CoV-2) in Shaanxi Province was reported on January 23, 2020. By March 26, 2020, a total of 245 confirmed infections with the original strain had been reported in the province [1]. Following the dynamic zero-COVID strategy [2] for preventing both imported and domestic coronavirus disease 2019 (COVID-19) infections, the SARS-CoV-2 outbreaks were successfully contained within several weeks in Shaanxi Province. From December 2020 to November 2022, more than 10,000 COVID-19 cases were reported by the Shaanxi Provincial Health Commission (http://sxwjw.shaanxi.gov.cn/). The leading SARS-CoV-2 variant causing domestic outbreaks changed from Delta between October 2021 and January 2022 to Omicron in March 2022. Omicron was less pathogenic but had greater infectivity than previous variants. Therefore, with high vaccine coverage, China began relaxing prevention and control measures at the end of November 2022, including the elimination of lockdown measures and mass nucleic acid testing, removing quarantine restrictions and health code requirements, and ceasing to identify close contacts. Subsequently, infections owing to the Omicron variant increased rapidly in the population. The peak of SARS-CoV-2 infections occurred between December 20 and 25, 2022, according to the Sentinel Community-Based Surveillance, but infections had declined to a low level by mid-January 2023 [3, 4]. Between December 2022 and January 2023, the most prevalent SARS-CoV-2 lineages involved in domestic cases were BA.5.2 and BF.7.14 [5].

Previous SARS-CoV-2 infection has been shown to be strongly protective against reinfection, but this protection can wane over time [6]. Additionally, evidence of increased SARS-CoV-2 reinfections owing to immune evasion has been reported as the Omicron variant continues to evolve under the humoral immune pressure exerted by vaccination or previous SARS-CoV-2 infection [7, 8]. China began managing COVID-19 as a Class B infectious disease, downgrading its classification from Class A. With the relaxation of COVID-19 prevention and control measures, epidemic waves of COVID-19 infection are more likely. Therefore, real-world data on reinfection rates are needed to elucidate the duration of immunity and the extent of cross-protection against emerging variants of concern.

In this study, we aimed to assess the incidence of SARS-CoV-2 reinfection during the epidemic period from December 2022 to February 2023, to determine the factors contributing to reinfection, and compare the clinical characteristics between first and second episodes of infection with different SARS-CoV-2 variants.

## Methods

### Study design

**Fig. 1:**
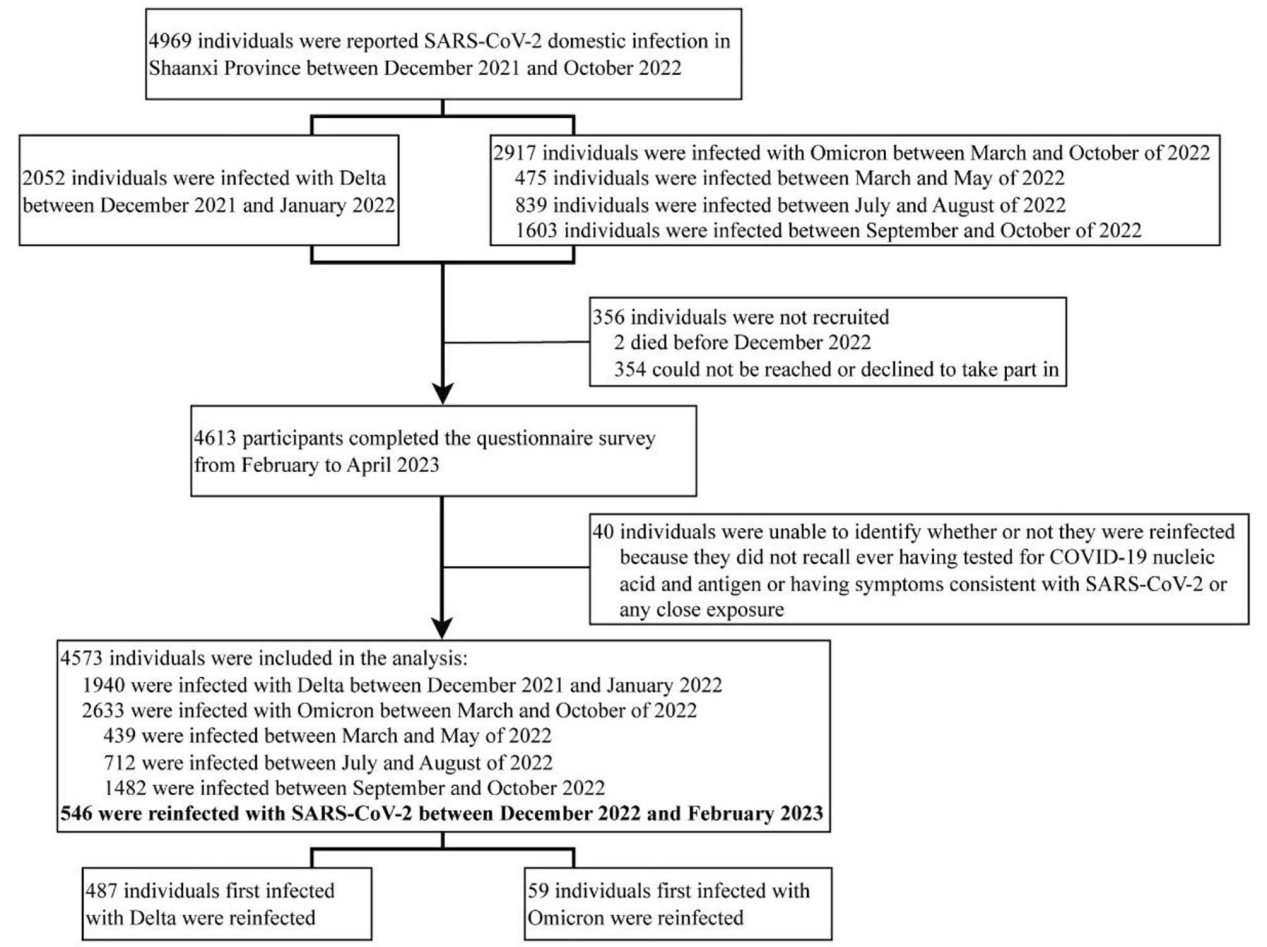
Study population

The cross-sectional study was conducted from February 2023 to April 2023. From December 8, 2021 to October 31, 2022, a total of 4969 cases of domestic SARS-CoV-2 infection were reported in Shaanxi province. Patients were categorized according to the variants causing the first infection. 2052 individuals were first infected with the Delta variant from 8 December 2021 to January 2022 and 2917 individuals were first infected with the Omicron variants from March 2022 to October 2022 (Fig. 1). Of those infected for the first time with the Omicron variants, 475 were infected between March and May of 2022, 839 were infected between July and August of 2022, and 1603 were infected between September and October of 2022. The diagnosis of patients’ first-infection was determined according to the National Diagnosis and Treatment Protocol for Novel Coronavirus Infection [9]. All infected individuals achieved clinical recovery from COVID-19 and obtained at least one negative result in reverse transcription polymerase chain reaction testing after their first infection.

### Data collection

Information on patient demographics, vaccination status, clinical symptoms and outcomes were collected via telephone surveys or face-to-face interviews by uniformly trained investigators from February 2023 to April 2023. Each of the 4969 study participants was first called to explain the purpose of the study. Each participant could choose whether to complete the questionnaire survey by telephone or in person. For participants under the age of 14 years or over age 75 years, questionnaires were completed by a parent or guardian over the phone or in person.

### Variables

In the study, SARS-CoV-2 reinfection was defined as a positive result on a SARS-CoV-2 nucleic acid or antigen test between December 2022 and February 2023 or meeting both of the following criteria: (1) presence of SARS-CoV-2 symptoms such as fever, cough, and others; and (2) members of the family, colleagues, friends, or other close contacts had tested positive in a SARS-CoV-2 nucleic acid or antigen test or had fever, cough, or other SARS-CoV-2-related symptoms.

Three types of COVID-19 vaccines were administered extensively in China: subunit vaccine, adenovirus vector vaccine, and inactivated vaccine. Except for the one-dose Ad5-nCoV and three-dose subunit vaccines, the immunization schedule for most vaccinations included two doses. Vaccination status was classified into five categories: unvaccinated, incomplete, complete, one booster dose, and two booster doses. “Complete” referred to a completed vaccination schedule. “Unvaccinated and Incomplete” referred to not having received any vaccinations and not completing the recommended vaccination schedule, respectively. “One or two booster doses” referred to completing the immunization schedule and receiving one or two additional doses of COVID-19 vaccine, respectively.

### Statistical analyses

All data were analyzed using IBM SPSS version 25.0 (IBM crop., Armonk, NY, USA) and Excel version 2020 (Microsoft Corporation, Redmond, WA, USA). Continuous and categorical variables are shown as numbers, percentages or medians, as appropriate. The Chi-squared test was used to compare the differences in proportion. Clinical symptoms of the first and second SARS-CoV-2 infections were compared, and logistic regression models were used to determine those factors contributing to a second infection. *P*-values ≤ 0.05 were considered statistically significant.

## Results

### Incidence of SARS-CoV-2 reinfection

**Fig. 2:**
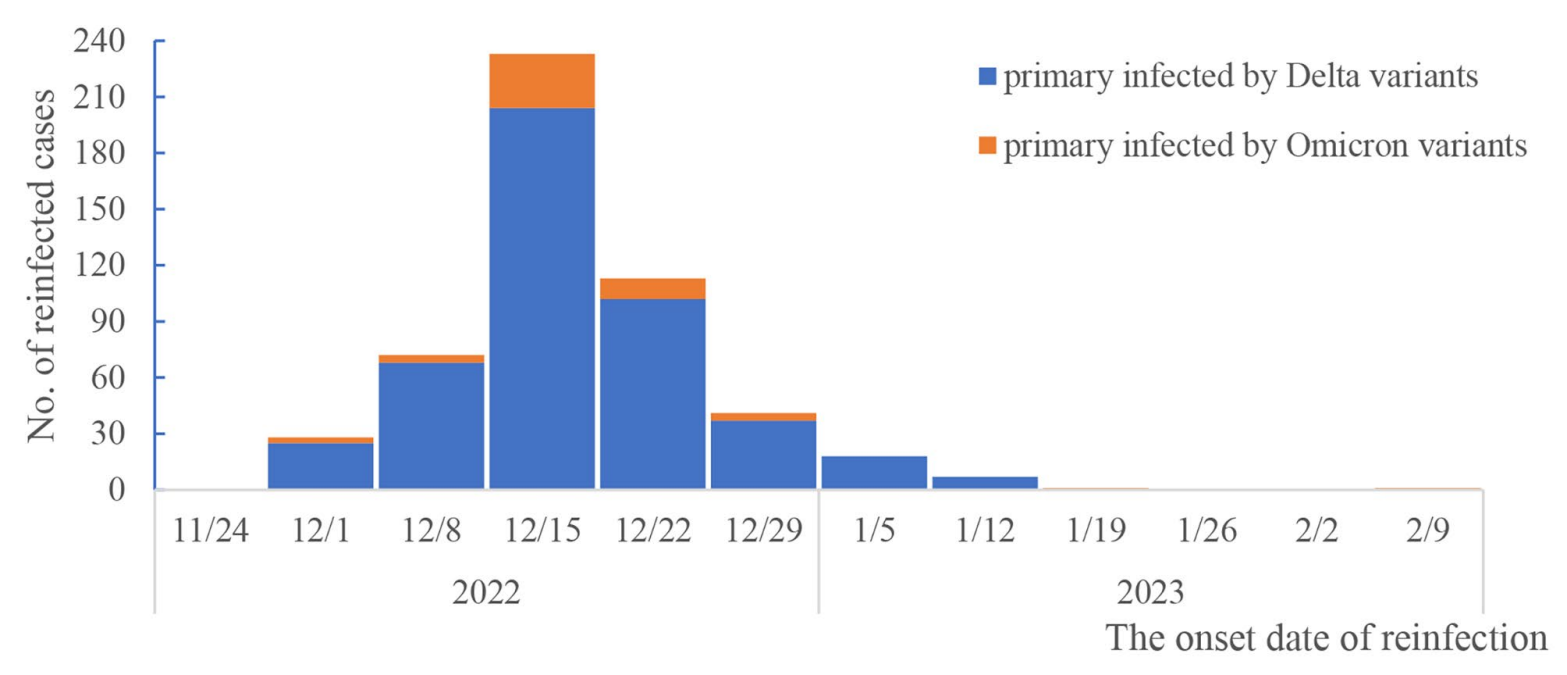
The incidence curve of SARS-CoV-2 reinfection over time

A total of 4573 participants were finally included in the analysis (Fig. 1), with 2390 male and 2183 female participants, ranging in age from 4 months to 96 years old. 1940 individuals were first infected with the Delta variant from December 2021 to January 2022, and 2633 individuals were first infected with the Omicron variants from March 2022 to October 2022. The demographic characteristics of the 4573 participants are presented in Table 1. Among study participants, 90.1% had completed primary vaccination against SARS-CoV-2, and 55.7% had received a booster vaccination. As for variants causing the first infection, 1940 individuals were first infected with Delta and 2633 individuals were first infected with Omicron. The distributions of sex and age were different between participants first infected with Delta and those first infected with Omicron- (χ^2^ = 4.674, *P* = 0.031 and χ^2^ = 43.595, *P* < 0.001, respectively). Overall, 546 SARS-CoV-2 reinfections were identified, with a pooled reinfection rate of 11.94% (546/4573, *95% CI*: 11.03–12.91). Reinfections peaked during the period December 15–22, 2022 (Fig. 2).

**Table 1:** Characteristics of the study population according to primary infected variants

Characteristic	Individuals primary infected with Delta (n, %)	Individuals primary infected with Omicron (n, %)	Total (n, %)
Gender			
Male	1050(54.1)	1340(50.9)	2390(52.3)
Female	890(45.9)	1293(49.1)	2183(47.7)
Age, years			
Median	35	37	36
<3	33(1.7)	52(2.0)	85(1.9)
3~17	210(10.8)	389(14.8)	599(13.1)
18~38	867(44.7)	943(35.8)	1810(39.6)
39~59	620(32.0)	895(34.0)	1515(33.1)
≥ 60	210(10.8)	354(13.4)	564(12.3)
Occupation			
Medical worker	47(2.4)	65(2.5)	112(2.4)
Other	1893(97.6)	2568(97.5)	4461(97.6)
Vaccination status			
Unvaccinated	167(8.6)	167(6.3)	334(7.3)
Incomplete	66(3.4)	53(2.0)	119(2.6)
Complete	751(38.7)	822(31.2)	1573(34.4)
One booster dose	947(48.8)	1506(57.2)	2453(53.6)
Two booster doses	9(0.5)	85(3.2)	94(2.1)
Interval since last vaccination			
Unvaccinated	167(8.6)	167(6.3)	334(7.3)
≤ 6 months	138(7.1)	155(5.9)	293(6.4)
6–12 months	669(34.5)	305(11.6)	974(21.3)
>12 months	966(49.8)	2006(76.2)	2972(65.0)
Reinfection status			
Non-reinfection	1453(74.9)	2574(97.8)	4027(88.1)
Reinfection	487(25.1)	59(2.2)	546(11.9)

Specifically, 487 reinfections were identified among those who were initially infected with the Delta variant, and 59 reinfections were identified among those initially infected with Omicron, when categorized according to the variant causing the first infection. The reinfection rate for the Delta variant was 25.10% (487/1940, 95% CI: 23.22%, 27.08%) 11–12 months after the primary infection. Reinfections rates for the Omicron variant were 1.28% (19/1482, 95% CI: 0.82–1.99), 1.96% (14/712, 95% CI: 1.18–3.27), and 5.92% (26/439, 95% CI: 4.07–8.54) 2–3 months, 4–5 months, and 7–9 months after the first infection, respectively. There was an increased reinfection rate over time (χ^2^_trends_ = 495.21, *P* < 0.001).

### Risk factors of SARS-CoV-2 reinfection

In the multivariate analysis of reinfection-related covariates (Table 2), for individuals who were first infected with the Delta variant, age (adjusted odds ratio [aOR] = 1.955, 95% CI: 1.331–2.871, *P* = 0.001) and sex (aOR = 1.249, 95% CI: 1.011–1.542, *P* = 0.039) were associated with reinfection. Compared with individuals over age 60 years, those aged 18–38 years were at higher risk of reinfection. However, the aOR (6.559, 95% CI: 2.878–14.951, *P* < 0.001) for people who were first infected with the Omicron variant revealed that being a medical worker posed an increased risk for reinfection. Logistic regression showed no evidence of an association between reinfection and the vaccination status, time interval since last vaccination, or comorbidities.

**Table 2:** Factors associated with reinfection according to primary infected variant

Factors	Delta			Omicron		
	Reinfection (n)	Univariate analysis OR (95% CI), P	Multivariate analysis OR (95% CI), P	Reinfection (n)	Univariate analysis OR (95% CI), P	Multivariate analysis OR (95% CI), P
Gender						
Male	245(23.3)	ref	ref	34(2.5)	ref	ref
Female	242(27.2)	1.227(0.999,1.507), 0.051	1.249(1.011,1.542), **0.039**	25(1.9)	0.757(0.449,1.277), 0.297	0.638(0.371,1.100), 0.106
Age, years(median ± SD)	33 ± 16.0			36 ± 13.3		
≥ 60	39(18.6)	ref	ref	5(1.4)	ref	ref
<17	49(20.2)	1.107(0.693,1.769), 0.669	1.016(0.628,1.643), 0.949	3(0.7)	0.478(0.113,2.014), 0.315	0.502(0.119,2.121), 0.349
18~38	269(31.0)	1.972(1.354,2.874), < 0.001	1.955(1.331,2.871), **0.001**	30(3.2)	2.294(0.883,5.959), 0.088	2.057(0.787,5.377), 0.141
39~59	130(21.0)	1.163(0.781,1.732), 0.456	1.166(0.779,1.542), 0.456	21(2.3)	1.677(0.627,4.483), 0.303	1.605(0.598,4.305), 0.348
Occupation						
Other	467(24.7)	ref	ref	51(2.0)	ref	ref
Medical worker	20(42.6)	2.262(1.257,4.071), 0.006	1.685(0.919,3.090), 0.092	8(12.3)	6.927(3.143,15.265), < 0.001	6.559(2.878,14.951), **< 0.001**
Vaccination status						
Unvaccinated & incomplete	58(24.9)	ref	ref	4(1.8)	ref	
Complete	199(26.5)	0.777(0.533,1.133), 0.190	0.711(0.471,1.073), 0.104	20(2.4)	0.998(0.313,3.179), 0.998	
≥One booster dose	230(24.1)	0.953(0.660,1.378), 0.800	0.783(0.530,1.159), 0.134	35(2.2)	0.914(0.324,2.575), 0.865	
Interval since last vaccination						
Unvaccinated	46(27.5)	ref	ref	4(2.4)	ref	
≤ 12 months	184(22.8)	0.611(0.354,1.053), 0.076	0.588(0.321,1.078), 0.086	11(2.4)	0.804(0.177,3.652), 0.778	
> 12 months	257(26.6)	0.895(0.626,1.279), 0.542	0.783(0.530,1.159), 0.222	44(2.2)	0.938(0.335,2.626), 0.903	
Comorbidities						
Have	-	-		5(2.1)	ref	
No	-	-		54(2.3)	1.060(0.420,2.676), 0.902	

Note: Age and sex were adjusted in Multivariate analysis. Bold *p* value represents statistical significance

### Clinical characteristics of SARS-CoV-2 reinfection

Out of the 546 reinfections identified, 73 cases were asymptomatic. Only two cases with a first infection owing to the Delta variant had a history of being hospitalized during the period of reinfection. Fever, cough, sore throat and fatigue were the four most common clinical symptoms (Table 3), reported in 470 participants during both first and second COVID-19 infections. We performed a comparative analysis of the clinical characteristics of reinfections among patients who were first infected with different variants. Remarkably, fever was less common during reinfections than during the initial infection among individuals first infected with Omicron (60.0% vs. 91.1%, *P* < 0.001). However, the asymptomatic rate was similar, and the incidence of other clinical symptoms did not differ significantly between the two episodes of infection. Ageusia (7.1% vs. 27.8%, *P* < 0.001), anosmia (5.7% vs. 27.6%, *P* < 0.001), and being asymptomatic (12.2% vs. 27.6%, *P* < 0.001) were all notably less common among individuals first infected with Delta variants during the reinfections than during their first infections.

**Table 3:** Comparison clinical symptoms of two SARS-CoV-2 infection episodes according to the primary infected variant

Clinical symptoms	Primary infected with Delta variants (*n* = 483)			Primary infected with Omicron variants (*n* = 59)		
	First infection (n, %)	Second infection (n, %)	χ2, P	First infection (n, %)	Second infection (n, %)	χ2, P
Fever	263(74.7)	290(68.4)	3.75,0.053	41(91.1)	27(60.0)	11.79,**0.001**
Cough	165(46.9)	211(49.8)	0.64,0.423	18(40.0)	19(42.2)	0.05.0.830
Sore throat	97(27.6)	130(30.7)	0.90,0.344	11(24.4)	12(26.7)	0.06,0.809
Fatigue	92(26.1)	118(27.8)	0.28,0.597	11(24.4)	12(26.7)	0.06,0.809
Coughing up phlegm	62(17.6)	72(17.0)	0.05,0.816	5(11.1)	8(17.8)	0.81,0.368
Myalgia	72(20.5)	76(17.9)	0.80,0.372	12(26.7)	17(37.8)	1.27,0.259
Nausea	62(17.6)	72(17.0)	0.05,0.816	6(13.3)	10(22.2)	1.22,0.270
runny nose	54(15.3)	66(15.6)	0.01,0.931	6(13.3)	10(22.2)	1.22,0.270
Headache	51(14.5)	56(13.2)	0.27,0.606	6(13.3)	6(13.3)	-
Ageusia	98(27.8)	30(7.1)	60.20, **< 0.001**	4(8.9)	3(6.7)	0.16,0.694
Anosmia	97(27.6)	24(5.7)	70.06, **< 0.001**	4(8.9)	2(4.4)	0.71,0.398
Asymptomatic	131(27.1)	59(12.2)	33.97, **< 0.001**	14(23.7)	14(23.7)	-

Note: Bold *p* value represents statistical significance

## Discussion

The prevalence of SARS-CoV-2 reinfection remained relatively low during the pre-Omicron period of the COVID-19 pandemic [10–11], but cases of reinfection rose substantially with the emergence of the Omicron variant, which quickly became dominant [7, 12–13]. Conducting the reinfection study is crucial to understanding the natural history of SARS-CoV-2 infection among Shaanxi provincial residents after China adjusted the anti-COVID measures. Having a knowledge of the proportions of reinfection is important when evaluating the impact on the healthcare system, allowing hospitals to stock up on supplies and medical equipment in advance of the outbreak. Characterizing the association between reinfection and vaccination history could provide information about the efficacy of hybrid immunization against Omicron. In our study, widespread SARS-CoV-2 reinfections occurred across the Shaanxi province during the first Omicron-dominant wave between December 2022 and February 2023. We found that the reinfection rate increased significantly over time, reaching a peak of more than 20% among those who were infected nearly one year prior to the Omicron period. However, within 6 months after being previously infected with Omicron, we found a low probability of reinfection.

Many studies on SARS-CoV-2 reinfection have been conducted in different various countries and regions during the different stages of the COVID-19 pandemic. Omicron was first reported in South Africa in early November 2021, sparking epidemics in over 100 countries and overcrowding hospitals in several of them. In comparison with our study, the rate of reinfection in Vojvodina, Serbia was substantially lower, with 5.49% of the study population experiencing reinfection between March 2020 and January 2022 and with a significantly increased incidence of reinfection during the Omicron wave [14]. Although lower than those of Guangdong province, China (28.3%) [15], the pooled reinfection rates in our study (11.94%) were comparable to those of Jiangsu province (12.61%) [16] and Zhejiang province (12.82%) [17]. The several local outbreaks in Shaanxi province were contained inside a specific geographical region throughout two maximum incubation periods owing to the dynamic zero-COVID strategy, which also prevented the outbreaks from spreading to nearby provinces and areas. Other Chinese provinces have effectively stopped the spread of viral infections by implementing the same prevention and control strategies. Because outbreaks involving different virus strains have occurred at different times throughout the county, the results of related studies have varied [15–17]. Nonetheless, the findings of past research show that individuals who were first infected with Omicron had a reduced rate of reinfection than those who were first infected with Delta or the original SARS-CoV-2 strain.

By the end of 2022, China was experiencing a sharp increase in COVID-19 infections. Following the relaxation of anti-COVID-19 measures, it is estimated that 82.4% of the country’s population was infected with Omicron between December 2022 and February 2023 [18]. Around December 25, 2022, the Chinese National Influenza Surveillance Network recorded a 60% positive rate [4]. During that time, SARS-CoV-2 was the predominant respiratory infectious disease in the nation. The rapid spread of infection put an undue burden on medical resources, making it difficult to acquire antigen testing reagents as well as causing a shortage of locations for nucleic acid testing in Shaanxi Province. As a result, certain COVID-19 infections could not be identified through antigen or nucleic acid testing. For this reason, we incorporated the following definition of reinfection in the survey: individuals with symptoms and an epidemiological history of SARS-CoV-2 infection but who failed to obtain a positive diagnostic result. Individuals who met these criteria were included in the study to ascertain whether reinfection had occurred.

Reinfections were more frequently reported among individuals between age 18 and 38 years who were first infected with Delta than among adults over age 60 years. This may be because younger people have more social contact and an increased likelihood of infection than older people. This group should be advised to use personal protection during subsequent epidemic waves. Similar to other studies [10, 15], medical workers were found to be more susceptible to reinfection than workers in other occupations. Medical personnel should implement efficient preventative measures at work because they have a significant risk of exposure to SARS-CoV-2.

According to published data, there were more asymptomatic carriers among Omicron-infected individuals than among those infected with other virus variants [19]. However, compared with the first infection episode, more patients who were previously infected with the Delta variant were more symptomatic. Many cities in Shaanxi Province implemented lockdown measures and carried out rounds of mass testing during the Delta outbreak that occurred between December 2021 and January 2022. With a positive SARS-CoV-2 nucleic acid test result, infected individuals were placed under quarantine and received prompt, efficient treatment. For this reason, more asymptomatic Delta infections were identified early on. Additionally, in accordance with a previously report [19], people infected with Omicron were less likely than those infected with Delta to experience a loss of smell or taste. Furthermore, BA.1 and BA.2 Omicron sublineages were responsible for several domestic infections prior to December 2022 in Shaanxi province, in the study we found the proportion of asymptomatic cases was comparable between first and second infection episodes among patients who were first infected with Omicron, which has not been previously reported.

We found the differences in clinical characteristics between first and second infections were small, and no correlation between vaccination and reinfection. In our study, more than 55% of the study population had received a booster dose of vaccine. Similarly, in a cohort study on the incidence of reinfection conducted in Shanghai, China, more than 60% of participants had received one booster dose. It also found that vaccination status was not significantly associated with reinfection [20]. Both studies pointed out the stronger capacity for vaccine breakthrough of emerging Omicron subvariants. Reinfection was not associated with an increased risk of COVID-19 hospitalization, as evidenced by our study that only two patients were hospitalized during their second infection in the study, which is consistent with the result of a systematic review [21]. However, this could be because of the study’s limited sample size in our study. Further research is needed to assess how well hybrid immunity can protect people aged 60 years or older or people with comorbidities.

The study has several limitations. First of all, because this was a retrospective study, an assessment of the SARS-CoV-2 symptoms and epidemiological exposure history in the study population identified nearly 40% of reinfections using a questionnaire survey but without a positive nucleic acid or antigen test. Therefore, recall bias could be present. Second, mass testing under China’s dynamic zero-COVID approach was replaced on December 8, 2022 by 10 New Prevention and Control Measures. Individuals were encouraged to purchase antigen test kits or undergo nucleic acid testing at a medical facility if deemed necessary. Thus, there may be an underestimation of reinfection in mild and asymptomatic cases, which can remain undetected if the patient does not visit a clinic or nucleic acid testing site. Third, a single variant was dominant during each local outbreak in Shaanxi province, and not all the infected patients underwent genotyping with whole genome sequencing, we were unable to compare the reinfection rates owing to various Omicron sub-variants. And patients’ serum antibody levels against COVID in Shaanxi province during December 2021 and October 2022 were insufficient to distinguish between reinfection based on the waning immunity and according to different SARS-CoV-2 subvariants.

## Conclusions

The present study highlights that the rates of SARS-CoV-2 reinfection among people who were previously infected with different variants in Shaanxi Province, China, increased over time during the Omicron-dominant epidemic wave between December 2022 and February 2023. Adults between 18 and 38 years and being a medical worker were associated with an increased risk of reinfection. To better shape our response and related policies, continued implementation of SARS-CoV-2 infection surveillance system and genome sequencing is recommended. Future investigations with extended follow-up will help to gain more understanding of the dynamics of SARS-CoV-2 reinfection.

## Data Availability

The datasets generated and analyzed during the current study are not publicly available so as preserve the respondents’ identity, but are available from the corresponding author on reasonable request.
